# Comprehensive Examination of Cholangiocarcinoma Patients Treated with Novel Targeted Therapies after Extended Molecular Profiling on Liquid Biopsies

**DOI:** 10.3390/cancers16040697

**Published:** 2024-02-06

**Authors:** Umair Mahmood, Elisya Muhamad Faizul, Sarah Howlett, Zahir Amin, Daniel Hochhauser, Kai-Keen Shiu, John Bridgewater, Khurum Khan

**Affiliations:** 1 Department of Gastrointestinal Oncology, University College Hospital NHS Foundation Trust (UCLH), London NW1 2BU, UK; umair.mahmood1@nhs.net (U.M.); sarah.howlett5@nhs.net (S.H.); daniel.hochhauser@nhs.net (D.H.); kaikeen.shiu@nhs.net (K.-K.S.); j.bridgewater@ucl.ac.uk (J.B.); 2 Faculty of Medical Sciences, University College London, London WC1E 6BT, UK; elisya.faizul.21@ucl.ac.uk; 3 Department of Radiology, University College Hospital NHS Foundation Trust (UCLH), London NW1 2BU, UK; zahir.amin@nhs.net; 4 University College London Cancer Institute, London WC1E 6DD, UK

**Keywords:** cholangiocarcinoma, targeted therapies, personalised medicine, liquid biopsy

## Abstract

**Simple Summary:**

Treatment with targeted agents is an emerging concept for cholangiocarcinoma, a rare cancer with a lack of effective therapies. However, there is limited real-world evidence regarding the use of targeted agents using molecular profiling data from liquid biopsies in clinical practice. This single-centre retrospective study aimed to characterise treatment efficacy and predictors of clinical benefit in cholangiocarcinoma following targeted therapies. Our findings suggest that patients with exposure to targeted agents and response to such treatment are the greatest beneficiaries of this personalized treatment approach; those with previous localised approaches such as surgery had further benefit, suggesting that localised treatment options can be considered in appropriate cases. Further research is needed to identify patients who are most likely to benefit from targeted agents using the liquid biopsy approach and evaluate the optimal sequence of such therapies to maximise treatment response and survival outcomes in this challenging clinical population.

**Abstract:**

Background: Cholangiocarcinoma (CCA) is associated with poor outcomes and limited treatment options, leading to increased use of targeted therapies for its management. Here, we performed one of the largest single-centre reviews evaluating outcomes following personalised targeted agents in CCA patients. Methods: All consecutive CCA patients receiving systemic therapy between January 2010 and April 2023 at UCLH were included. The primary objective of this study was to evaluate treatment response, survival outcomes and predictors of clinical benefit in CCA patients treated with molecularly guided therapies. Patient demographic factors, disease characteristics and survival outcomes were evaluated using the Kaplan–Meier method and Cox proportional-hazards models. Results: Of the 227 consecutive CCA patients, 162 (71%) had molecular profiling, of whom 56 (35%) were eligible and 55 received molecular-targeted treatment. CCA histological classifications comprised intrahepatic (N = 32), extrahepatic (N = 11), hilar (N = 4) and unknown (N = 9) subtypes. Most patients received targeted agents based on genomic profiling in a second treatment line setting (N = 34). Frequently observed genomic alterations occurred in the *FGFR2* (N = 21), *IDH1* (N = 7) and *BRCA2* (N = 6) genes. Median progression-free survival (PFS) following first-, second- and third-line systemic therapy and overall survival (OS) were 8.44 (95% CI, 7.49–12.78), 5.65 (95% CI, 3.71–7.13), 5.55 (2.79–12.58) and 29.01 (24.21–42.91) months, respectively. CCA subtype and FGFR/BRCA molecular aberration status were not associated with PFS or OS. However, a prior CCA-related surgical history was predictive of OS (*p* = 0.02). Stratification by best overall response to second-line targeted agents demonstrated an association with PFS (*p* = 0.002) and OS (*p* = 0.02). Duration of treatment with second-line targeted therapy was associated with OS (*p* < 0.001). Conclusions: Patients receiving targeted therapeutics achieved promising outcomes, especially those attaining a favourable treatment response and those receiving targeted agents for longer periods. Liquid biopsies can reliably provide information on extended molecular profiling to aid patient selection for personalised therapies.

## 1. Introduction

Cholangiocarcinoma (CCA) is a less common but well-established cancer type representing the second most common primary liver cancer worldwide [1]. Surgery is the primary treatment option for localized tumours, but only a small cohort of patients is eligible for this intervention. Systemic treatment options are currently limited, comprising primarily gemcitabine, cisplatin and immunotherapy [2,3] for patients with locally advanced or metastatic cholangiocarcinoma in a first-line setting [4].

Treatment with targeted agents provides another possible opportunity to ensure optimal management of this challenging clinical condition. For instance, targeting the aberrant Fibroblast Growth Factor (FGF) signalling pathway, which is implicated in intrahepatic cholangiocarcinoma, has been an active area of clinical investigation. Currently, pemigatinib, which inhibits Fibroblast Growth Factor Receptor 1 (FGFR1), FGFR2 and FGFR3, as well as ivosidenib, which targets mutant isocitrate dehydrogenase-1 (IDH1), remain the only targeted agents approved by the European Medicines Agency for the treatment of CCA following the promising findings of the Phase II FIGHT-202 and ClarIDHy trials [1,5,6]. However, there is a paucity of data regarding predictors of clinical benefit and treatment outcomes following targeted agents among CCA patients. It also remains to be seen whether a combined treatment approach with the addition of chemotherapeutic regimens or immune checkpoint inhibitors (ICIs) can translate into improved response rates and survival outcomes.

We conducted the largest single-centre review to date of patients with CCA managed with targeted agents with or without chemotherapy/immunotherapy. The primary objective of this study was to assess clinical outcomes comprising treatment response, duration of clinical benefit, progression-free survival (PFS) and overall survival (OS) in this emerging clinical population. We also aimed to identify clinicopathological variables associated with PFS and OS, which could help refine patient selection to optimise treatment for CCA patients being selected for systemic therapy. One of the aims of this study was to define groups of patients who would derive the greatest therapeutic benefit from targeted agents. Identification of such patients is important given the potential for long-term benefit and reduced side effects of targeted agents (and ICIs) as compared to other chemotherapeutic regimens, allowing patients to continue such treatments to ensure a greater duration of treatment benefit.

## 2. Material and Methods

### 2.1. Patient Selection

Patients were eligible for our study if they were ≥8 years of age with a diagnosis of CCA and were treated with any targeted agent from January 2010 to April 2023 at the University College London NHS Foundation Trust. Since our study pertained to patients receiving targeted agents, individuals receiving only chemotherapy or radiation were excluded. Patients receiving systemic treatment as part of an ongoing clinical trial were also excluded since their outcome data could change before the final results are published. 

We abstracted data from patient medical records including clinician’s notes, radiology, radiation oncology, operative and pathology reports, and clinifcal reports regarding molecular alterations and microsatellite instability levels. A microsatellite refers to a short, repeated sequence of DNA [7]. An alteration in these repeated DNA sequences due to mutational events leads to a cancer state called microsatellite instability, which can affect cancer prognosis and treatment. Molecular profiling using next-generation sequencing (NGS) was performed using circulating tumour DNA (ctDNA) from blood samples in institutional laboratories as well as via commercial platforms comprising FoundationOne^®^ Liquid CDx and Guardant360^®^.

### 2.2. Statistical Considerations

Primary outcome measures included PFS following 1st-, 2nd- and 3rd-line systemic treatment. PFS was defined as the time between the first dose of systemic agent till radiological/clinical evidence of disease progression, date of last follow-up or death. We also evaluated OS in our complete cohort. 

The overall response rate (ORR) was assessed by the RECIST v1.1 criteria as detailed in Supplementary Table S1. Clinical benefit was defined as any evidence of stable disease (SD), partial response (PR) and complete response (CR) during systemic therapy, whilst the duration of response was determined by comparing the date of these first radiographic outcomes with the date of radiographic progression, also based on the RECIST v1.1 criteria. 

Median and interquartile range were used to summarise the continuous variables of age, duration of treatment with targeted therapy and duration of clinical benefit. Frequency tables summarised categorical variables including gender, smoking status, cholangiocarcinoma subtype, stage at initial diagnosis, anatomic site of metastasis, prior treatment, type of systemic therapies received by patients and best overall response to each systemic therapy. Outcomes were analysed using Kaplan–Meier actuarial survival methods and Cox proportional-hazards models (CoxPH). Analysis was also performed using univariate and multivariate methods. Statistical significance was defined as *p* < 0.05 using log-rank tests to identify associations between clinical and treatment variables with PFS and OS. All data analysis was performed using R (version 4.2.3) in RStudio (version 2022.12.0.353) and Microsoft Excel (version 16.45) software. 

## 3. Results

### 3.1. Baseline Characteristics

**Figure 1:**
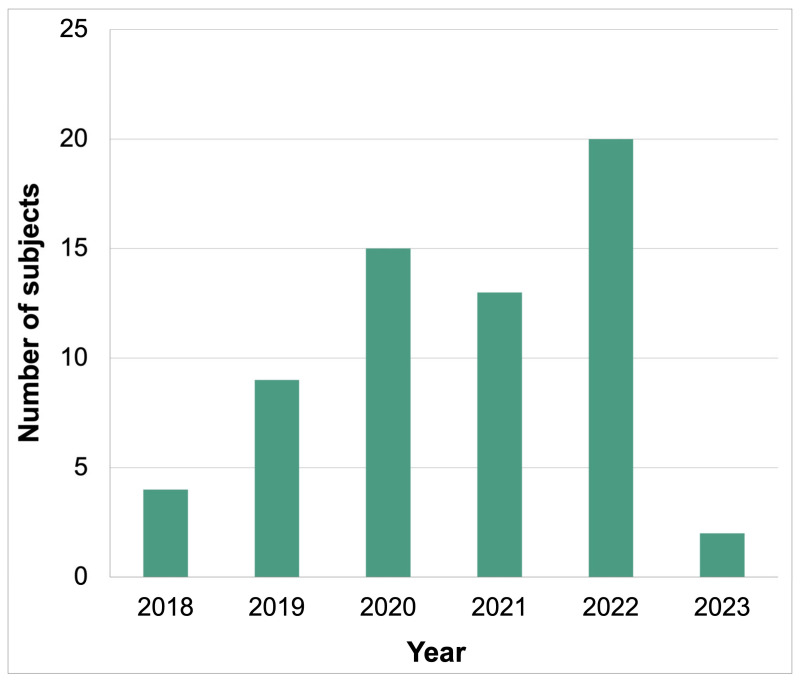
Annual distribution demonstrated an increased use of targeted agents for the treatment of cholangiocarcinoma in recent years.

We screened 227 patients and identified 56 (35%) eligible subjects who received targeted therapy for cholangiocarcinoma. The median age at CCA diagnosis was 59 and the majority of patients were female (N = 33, 59%) and non-smokers (N = 13, 23%) (Table 1). Patients whose performance status was available prior to the first targeted therapy mostly had an Eastern Cooperative Oncology Group Performance Status of 1 (N = 18, 32%), with hypertension as the most common co-morbidity (N = 11, 20%). Patients were classified under the following CCA histological subtypes: intrahepatic (N = 32), extrahepatic (N = 11), hilar (N = 4) and unknown (N = 9). The incidence of localised vs. metastatic disease presentation was equivalent in our cohort of patients at the time of CCA diagnosis (N = 26, 46%) (Table 1). A significant number of subjects in our cohort was previously managed surgically (N = 23, 41%) (Table 1). However, an increasing number of patients was treated with targeted therapies in more recent years, except for the 2020–2021 period, likely due to the impact of the COVID-19 pandemic, and in 2023 due to the conclusion of data collection up until April 2023 (Figure 1). We evaluated our patients up to six lines of systemic therapy (Table 2). The vast majority of our patients received chemotherapy as their first line of systemic treatment, which mainly comprised cisplatin and gemcitabine (N = 38, 68%) (Table 2, Supplementary Table S2). Most subjects received targeted monotherapy in a second-line setting, which mainly consisted of futibatinib (N = 11, 20%) (Table 2, Supplementary Table S2).

### 3.2. Molecular Analysis

**Figure 2:**
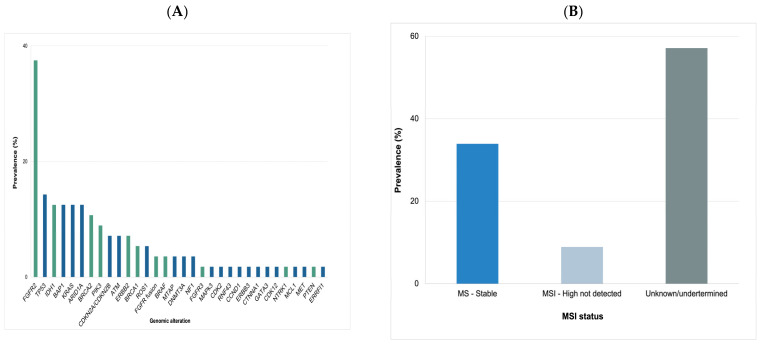
The landscape of genomic alterations (**A**) and microsatellite instability (MSI) status (**B**) in evaluated patients with cholangiocarcinoma.

Among the 227 patients who were screened in our study, 162 (71%) subjects underwent ctDNA-based molecular profiling using institutional and commercial next-generation sequencing platforms. Among the 56 eligible subjects in our study, the most common clinically actionable molecular aberrations comprised alterations in the *FGFR2* (N = 21), *IDH1* (N = 7) and *BRCA2* (N = 6) genes (Figure 2A). Many patients (N = 19) had a microsatellite-stable status (Figure 2B). In our cohort of selected patients, 55 subjects received treatment based on their molecular profiling results. Patients in the second-line treatment setting formed the largest group of subjects to receive molecularly guided targeted treatment (n = 32, 91%) (Table 2).

Stratification of PFS outcomes by molecular status did not reveal any associations with respect to FGFR (*p* = 0.05) and BRCA molecular aberrations (*p* = 0.25) following first-line therapy (Table 3). Molecular aberrations such as FGFR (*p* = 0.34) and BRCA status (*p* = 0.20) also lacked an association with PFS after treatment with the second line of systemic agents (Table 3). We did not note an association between PFS and FGFR aberrations (*p* = 0.51) in a third-line setting; however, PFS was associated with pathogenic BRCA variants in the univariate analysis (*p* = 0.02) but not in the adjusted models (*p* = 0.07) (Table 3). Finally, a lack of association was observed between OS and FGFR (*p* = 0.52) as well as BRCA mutational status (*p* = 0.38) (Supplementary Table S3).

### 3.3. Efficacy

Treatment response and survival outcomes following first-line systemic therapy

In the first-line treatment setting, chemotherapy being the predominant choice of systemic therapy did not appear to be effective in disease control as evidenced by a 2% complete response rate (n = 1) and a 23% partial response rate (n = 13) (Supplementary Table S4). The vast majority of patients (n = 15, 27%) continued to develop disease progression despite treatment with chemotherapeutic agents. The median duration of clinical benefit (DOCB) was limited to 5.42 months (interquartile range (IQR) = 3.22–6.31 months), which remained similar for patients receiving cisplatin and gemcitabine (median DOCB = 5.42 months, IQR = 3.02–8.08 months) (Supplementary Figure S1A). 

**Figure 3:**
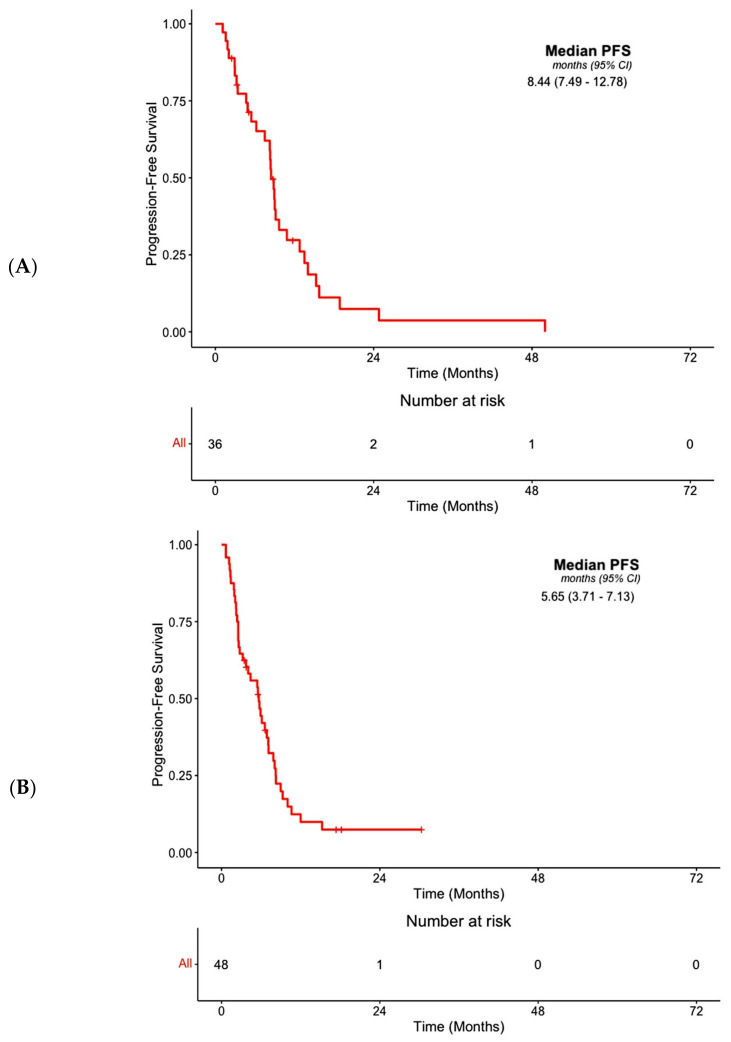

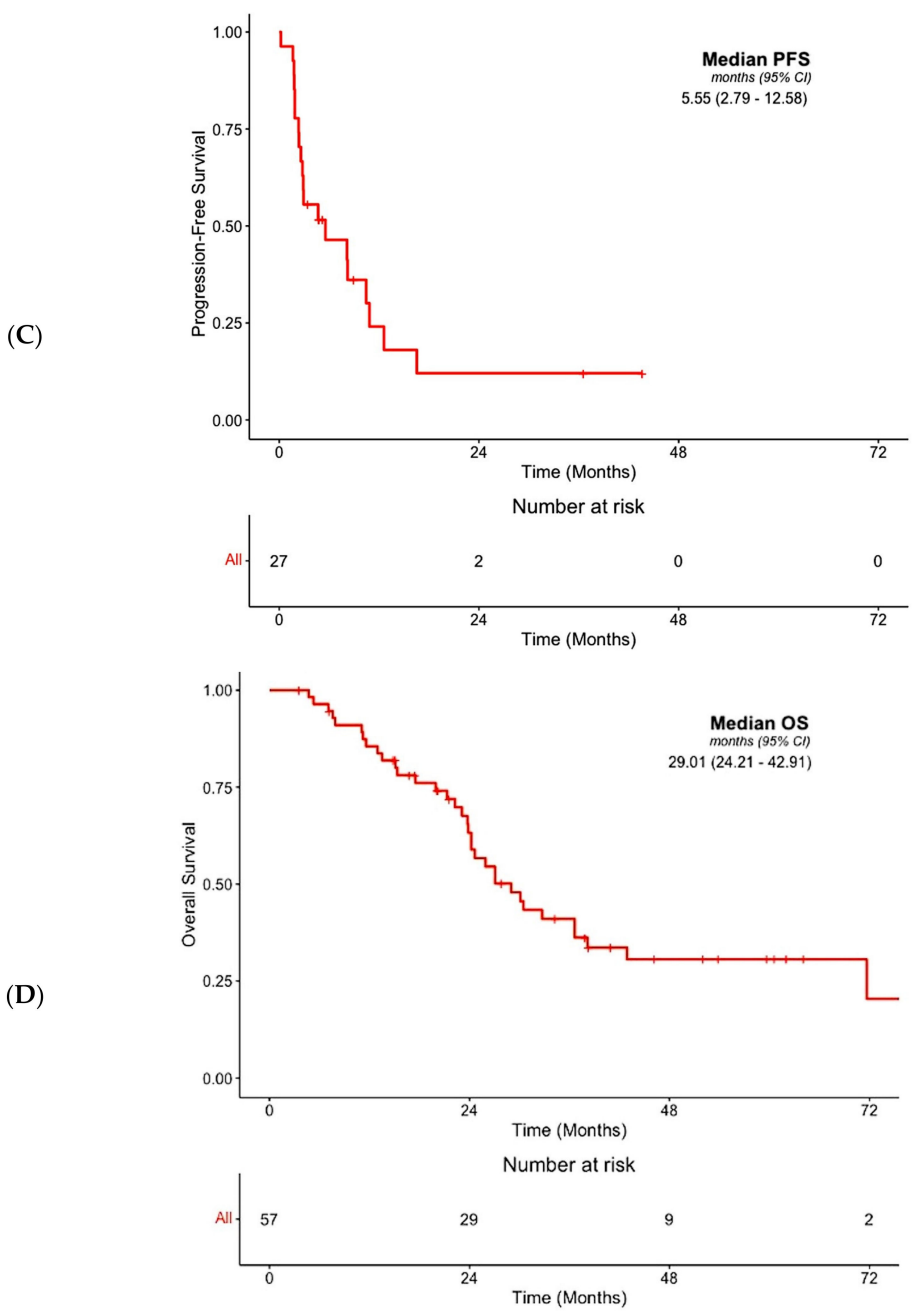
Outcomes following treatment with systemic agents are depicted by PFS based on the first progression event at any site following treatment with any 1st-line systemic agent (**A**), 2nd–line systemic agent (**B**) or 3rd–line systemic agent (**C**). OS outcomes following treatment with systemic agents for the entire cohort are also demonstrated (**D**).

The median PFS following first-line systemic therapy was 8.44 months (95% CI, 7.49–12.78) (Figure 3A). Univariate analysis did not demonstrate any association between PFS and age (*p* = 0.13), gender (*p* = 0.82) or smoking history (*p* = 0.38) (Table 3). We also did not observe any association between PFS and CCA histological classifications including intrahepatic (*p* = 0.70) and extrahepatic subtypes (*p* = 0.53) (Table 3). Additionally, patients with a prior surgical history for the treatment of CCA were not likely to attain better PFS outcomes as compared to their non-surgical counterparts (*p* = 0.96) (Table 3).

Treatment response and survival outcomes following second-line systemic therapy

Within a second-line systemic treatment setting, response rates remained low among the chemotherapy cohort with patients only achieving a partial response rate limited to 9% (n = 5) (Supplementary Table S5). Subjects receiving targeted therapies had a partial response rate of 18% (n = 10), but an elevated rate of progressive disease at 27% (n = 15) was also noted in this cohort of patients (Supplementary Table S5). However, patients on targeted therapeutics had a prominently higher median duration of clinical benefit of 4.17 months (IQR = 2.33–8.18 months) compared to patients receiving chemotherapy (median DOCB = 2.99 months, IQR = 2.66–3.91 months) (Supplementary Figure S1B). Most patients in the targeted therapy cohort had received futibatinib, which derived the greatest clinical benefit in this instance as noted by a median DOCB of 5.60 months (IQR = 2.11–7.35 months) (Supplementary Figure S1B). The second most frequently administered targeted agent was regorafenib in a second-line setting where all except one patient had progressive disease as their best overall response. 

**Figure 4:**
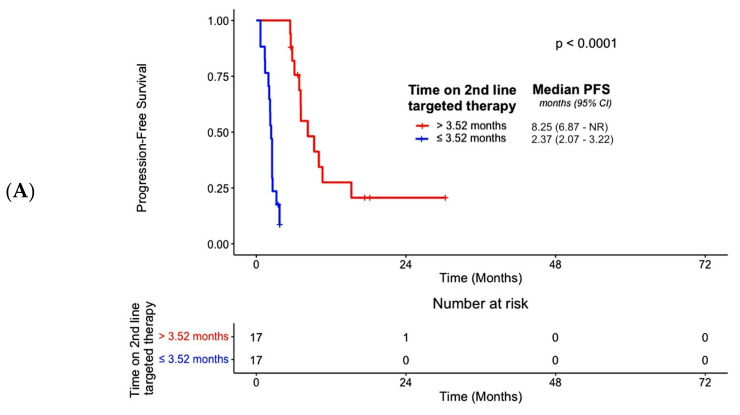

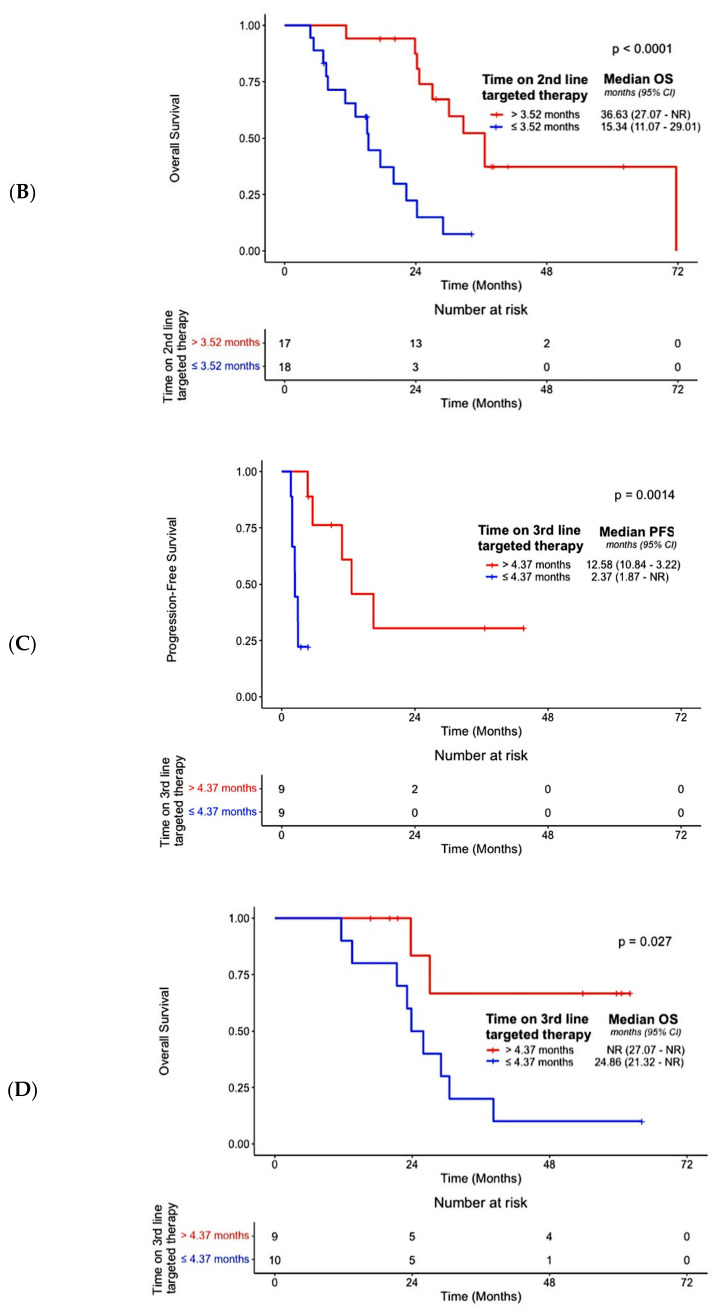
Associations between time on 2nd–line targeted therapy and PFS (**A**) and OS (**B**), respectively. The median value of 3.52 months was used to divide subjects. Associations were also evaluated between time on 3rd–line of targeted agents and PFS (**C**) and OS (**D**), respectively. In this instance, the median value of 4.37 months was used to stratify subjects.

The median PFS for all patients on second-line therapy was 5.65 months (95% CI, 3.71–7.13) (Figure 3B). There were no associations between PFS and age (*p* = 0.20), gender (*p* = 0.61) or smoking history (*p* = 0.91) (Table 3). Similarly, there were no associations between PFS and intrahepatic (*p* = 0.55) and extrahepatic subtypes (*p* = 0.35) (Table 3). PFS was also not associated with prior surgical history for the management of CCA (*p* = 0.86) (Table 3). However, PFS was associated with the best overall response on second-line targeted therapy (*p* < 0.001), which remained significant after adjustment for co-variates (*p* = 0.002) (Supplementary Figure S2A, Table 3). We also observed an association between OS and best overall response on second-line targeted therapy (*p* = 0.0091), which also remained significant in multivariate analysis (*p* = 0.02) (Supplementary Figure S2B, Supplementary Table S3). Moreover, patients receiving futibatinib had an improved PFS (*p* = 0.004) after adjustment for co-variates but not OS (*p* = 0.54) than subjects receiving other forms of targeted therapy (Supplementary Figure S3A,B, Supplementary Table S3). We also noted that subjects receiving second-line targeted therapy for a period greater than the median duration of 3.52 months experienced prolongation of their PFS (*p* < 0.0001) in univariate analysis, as well as OS (*p* < 0.001) in the adjusted model as compared to individuals receiving targeted therapy for ≤3.52 months (Figure 4A,B, Table 3, Supplementary Table S3). 

Treatment response and survival outcomes following third-line systemic therapy

Patients receiving targeted therapeutics in a third-line setting had a partial response, serving as the best overall response for most of this cohort, of 24% (n = 7) (Supplementary Table S6). Such patients also had a greater median DOCB of 7.03 months (IQR = 2.68–12.29 months) compared to patients in the chemotherapy group (median DOCB = 5.03 months, IQR = 2.63–5.42 months) (Supplementary Figure S1C). Most of the patients had also received futibatinib as their choice of third-line therapy, where an extension of median DOCB was observed at 10.09 months (IQR = 4.71–13.17 months). The second most frequently administered targeted agent was regorafenib in a third-line setting where all except two subjects had progressive disease as their best overall response. 

The median PFS for all patients on third-line therapy was 5.55 months (2.79–12.58) (Figure 3C). An association between PFS and age (*p* = 0.93), gender (*p* = 0.75) and smoking history (*p* = 0.82) was not observed (Table 3). We did not note an association between PFS and intrahepatic (*p* = 0.16) or extrahepatic CCA subtypes (*p* = 0.09) (Table 3). Prior surgical management of CCA (*p* = 0.81) was not associated with PFS (Table 3). However, PFS was associated with the best response on third-line targeted therapy (*p* = 0.02), which remained significant after adjustment for co-variates (*p* = 0.02) (Supplementary Figure S2C, Table 3). We did not observe an association between OS and the best response on third-line targeted therapy (*p* = 0.36) (Supplementary Figure S2D, Supplementary Table S3). Subjects receiving futibatinib did not experience better PFS (*p* = 0.40) or OS (*p* = 0.94) outcomes compared to patients receiving other types of targeted therapies (Supplementary Figure S3C,D, Table 3, Supplementary Table S3). However, there was an association between the median duration of third-line targeted therapy exceeding 4.37 months and PFS (*p* = 0.0014) as well as OS (*p* = 0.027) (Figure 4C,D), and this association remained significant only for PFS in adjusted models (*p* = 0.01) (Table 3). Lastly, we noted an association between OS and prior CCA-related surgical history (*p* = 0.01), which remained significant after adjustment for co-variates (*p* = 0.02) (Supplementary Table S3). The median overall survival for the entire cohort was 29.01 months (24.21–42.91) (Figure 3D). 

Exceptional responders on targeted agents

We observed three subjects who attained an exceptional treatment response and survival outcomes following clinical management of their underlying CCA with targeted agents. Subject # 6 had previously failed first-line treatment on cisplatin and gemcitabine after 3 months of therapy. She was noted to have an FGFR2 rearrangement in intron 17 and subsequently received second-line futibatinib, which led to a durable PR lasting for over 27 months (Supplementary Figure S4). The patient is currently alive and has not experienced treatment recurrence at the time of last follow-up. 

**Figure 5:**
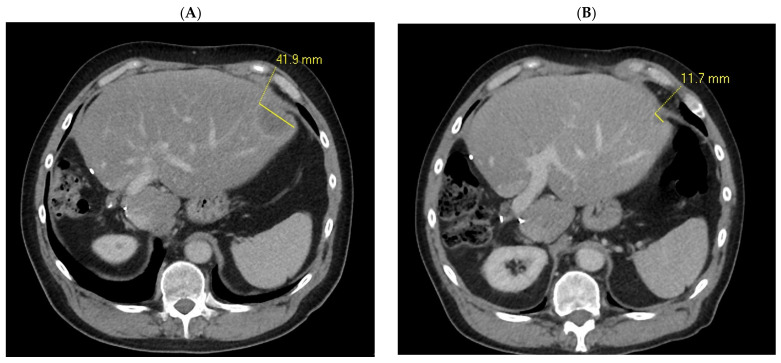
Pre-treatment baseline scan of Subject #42 from April 2019 demonstrates 1 liver metastasis with largest axial measurement of 41.9 mm (**A**). Patient achieved partial response in May 2019 while on 3rd-line futibatinib, which demonstrated the greatest regression in liver tumour volume in August 2019 limited to 11.7 mm (**B**).

Patient # 42 with an FGFR2-BICC1 fusion developed a durable PR lasting over 9 months following treatment with third-line futibatinib (Figure 5). This patient also had prior surgery for intrahepatic CCA with R0 resection status, which could have possibly contributed to his excellent PFS lasting 10.84 months and extension of OS to 60.62 months.

Subject # 46 had previously failed treatments comprising cisplatin and gemcitabine, folinic acid, fluorouracil and oxaliplatin (FOLFOX regimen), as well as regorafenib, with progressive disease as her best response on these treatments. She subsequently developed an FGFR2-DOCK1 fusion, which was detected on a commercial liquid biopsy platform that led to the assignment of futibatinib in a fourth-line setting, resulting in a brief (2 months) but profound PR (Supplementary Figure S5). 

## 4. Discussion

CCA, whether locally advanced or metastatic, represents an important avenue of ongoing clinical investigations to bridge a major unmet clinical need via the development of novel, efficacious therapies with a high safety profile. We endeavoured to evaluate clinical outcomes following targeted therapeutics among such patients since data pertaining to this patient population are currently lacking. To our knowledge, this retrospective review represents the largest examination of patients with CCA who were treated with systemic targeted agents driven by molecular profiling results.

The ABC-06 trial demonstrated a relatively modest efficacy of FOLFOX in patients refractory to first-line gemcitabine and cisplatin treatment; however, the trial expanded the scope of second-line therapies in CCA. Our data highlight the importance of targeted therapies in appropriately selected patients in a second-line setting as we observed better PFS and OS compared to that demonstrated by FOLFOX in ABC-06 [8]. Moreover, despite treatment with multiple lines of systemic therapy, OS outcomes for our cohort of patients appeared to be over twice as high as in patients receiving gemcitabine, cisplatin and durvalumab in a first-line setting [3]. Hence, our data suggest that targeted intervention offers a viable alternative to systemic management of patients with locally advanced or metastatic cholangiocarcinoma given the limited survival benefit associated with chemotherapy use. 

Cross-comparison of our patients receiving FGFR inhibitors in a second-line targeted setting with subjects on the FIGHT-202 trial lends further credence to this therapeutic management strategy as noted by improved survival outcomes conferred with a targeted treatment approach. Here, all of our subjects, with the exception of two cases, had FGFR2 fusions/alterations, and comparison of such patients demonstrates similarities in a median PFS of 6.75 months vs. 7.10 months in the FIGHT-202 trial [9]. However, we did note a higher OS of 23.85 months in our cohort as compared to the median OS of 17.50 months in that Phase II trial [9]. This could possibly be due to differences in prior lines of systemic treatment, where 39% of patients in FIGHT-202 with FGFR2 fusions or rearrangements had received two or more previous therapies [5], leading to increased treatment resistance, whereas our OS findings pertain to all subjects in a second-line targeted treatment setting. Additionally, an OS difference could also be attributed to the difference in rates of patients with metastatic disease volume. In our cohort, 47% of patients had metastasis at the time of second-line targeted treatment as compared to 82% in the FIGHT-202 trial [5]. The rates of patients with lung and bone metastases in our cohort were 20% and 13%, respectively, as compared to the higher rates of 94% and 20% in the FIGHT-202 trial [5]. As observed in our study as well, high treatment discontinuation rates driven primarily by progression on pemigatinib seem to be a challenge, possibly driven by polyclonal secondary FGFR2 mutations as a clinical resistance mechanism [9,10]. Additionally, findings from the recent FOENIX-CCA2 trial involving the treatment of CCA with futibatinib demonstrated a comparable median OS of 21.7 months, as well as a slightly higher median PFS of 9.0 months as reported by the trial investigators [11]. Likewise, IDH inhibitors have yielded success in terms of improving outcomes for CCA patients in a second-line setting, with median PFS and OS limited to 2.7 and 10.3 months, respectively [6,12]. Similar to chemotherapy, an added challenge of targeted agent use is the high yet variable rate of toxicity. For instance, the rate of commonly observed > grade 3 treatment-emergent adverse events for anti-FGFRs can range from 69% to 80% [9,13]. The clinical benefit in terms of improved treatment response and survival with targeted agent use is limited to 47% of patients who harbour an actionable mutation [14], with only 14% of intrahepatic CCA patients possessing an FGFR2 protein alteration [11] and thus eligible for anti-FGFR therapies for intrahepatic CCA. However, considering the current lack of efficacious therapies, our data along with results from the FIGHT-202 and FOENIX-CCA2 trials taken together demonstrate that targeting FGFR alterations might be the most prudent therapeutic approach in select CCA patients.

The recent advent of liquid biopsies continues to shape the clinical management of patients with gastrointestinal malignancies. Their current utility has ranged from acting as a highly sensitive method for the detection of somatic variants in subjects with colonic aberrations [15] to serving as a negative prognostic biomarker in gastro-oesophageal cancer [16] and metastatic colorectal cancer [17]. Emerging evidence in the context of CCA has successfully demonstrated the detection of clonal and polyclonal gatekeeper mutations following anti-FGFR therapy using liquid biopsy techniques [10,18]. Recent investigations also suggest the practical utility of using this minimally invasive and cost-effective technique for diagnosing CCA in high-risk populations (with primary sclerosing cholangitis) and the prognostic stratification of patients with CCA [19]. These findings, along with our data, lend credence to the notion that liquid biopsies can serve as an effective and serially repeatable method for CCA screening, therapeutic monitoring and identification of specific mutations following treatment recurrence. Such recurrence following targeted agents can aid in the identification of resistant but potentially druggable mutations in evolved tumours following selective pressure exerted by these therapies and can guide treatment selection for the next line of targeted agents. However, certain limitations surrounding liquid biopsy techniques employing ctDNA methods need to be overcome before the current gold standard of tumour specimen sequencing can be replaced by such techniques. For instance, the diagnostic utility of ctDNA is limited in patients with early-stage cancer (but not metastatic disease) due to low concentrations of ctDNA in blood plasma, which can decrease the analytical sensitivity of the assay and pose a challenge to the detection of disease relapse [20,21]. Detection of a broad panel of mutations with low ctDNA levels is possible but is more resource-intensive [20]. Such methods can often detect mutations that are not always relevant to cancer biology and cannot be always actioned due to the lack of available targeted therapies [21]. Other factors currently limiting the integration of liquid biopsies in a centralised clinical setting involve significant financial investment to procure technical resources, a lack of trained staff collecting samples with an awareness of assay protocols and preanalytical issues that might affect assay performance, as well as a lack of a molecular board to help interpret genomic data [21]. These issues can be partly addressed via a decentralised commercial platform, which can provide practical solutions due to the presence of an experienced molecular board and reduced profiling costs resulting from economies of scale [21]. Therefore, given the limited outcomes associated with standard treatments in a metastatic setting [4] and the plethora of druggable molecular targets in CCA, we propose the incorporation of tumour specimen sequencing with a later shift towards improved liquid biopsy-based molecular profiling techniques in future standard clinical practice prior to treatment initiation and during the course of systemic therapy administration. 

Our study is limited by heterogeneity including CCA subtype and anatomic site of metastases, as well as variability in systemic treatment types, doses and schedules, which would have affected response and survival outcomes. Treatment response and disease stabilisation rates in our study were fairly modest among patients treated with targeted agents, possibly due to the prescription of different agents with varying mechanisms of action and efficacies. Variability in the expression of actionable molecular targets among different CCA subtypes [22] could have contributed to overall modest response rates in second- and third-line settings. We noted a reduction in the number of patients who were treated with targeted agents during the COVID-19 pandemic. The pandemic caused difficulties with timely access to clinical teams, molecular testing and radiographic scans due to dynamic variations in clinical workflow and logistics surrounding treatment management. Although we adjusted our analysis with select variables, the presence of additional confounders such as a mixed clinical population with both localised and metastatic disease as well as a varying anatomic distribution of metastatic deposits could have influenced treatment outcomes in our study. We also did not evaluate any pre-treatment and post-treatment changes by biopsying tumour specimens. It is possible that differences in treatment response in our patient population could be attributed to the heterogeneity in the tumour microenvironment (TME) that might represent a tumour-promoting milieu or an anti-tumour, pro-immunogenic environment. Patient referral to cancer specialists can lead to improved survival outcomes compared to the underlying clinical population due to the exclusion of patients who are frail or unable to travel to the treatment centre due to socioeconomic reasons [23]. This possible selection bias could have impacted the outcomes observed in our study. There were also limited numbers of subjects within subgroups, which may have resulted in suboptimal statistical power for some of the subtype analyses with survival outcomes. The retrospective nature of our study with small subgroup sizes warrants further validation of observations using larger, prospectively designed studies. 

Despite the presence of advanced disease, we noted a subset of our patients who attained significant and durable responses in second- and third-line treatment settings. This clinical benefit can be mainly attributed to selective treatment with futibatinib among patients with FGFR2 aberrations leading to the effective abrogation of FGFR-dependent downstream signalling, which promotes cellular proliferation and survival, resulting in potent anti-tumour activity. Among the eight patients receiving second-line futibatinib with available DOCB data, we noted five patients who did not have any pre-existing co-morbidities out of whom three achieved PR and two attained SD. Thus, increased overall physical fitness owing to a lack of co-morbidities could have promoted improved treatment tolerance and translated into improved response outcomes in these patients. We also noted a set of subjects attaining an exceptional response to targeted agents. However, due to the paucity of current data on this orphan disease, the role of genomic aberrations in explaining the exceptional response to futibatinib remains unclear. However, we can speculate on prior treatment history, which could have influenced these outcomes. For instance, surgery and chemotherapy could have reduced the tumour burden, especially involving cancer subclones that are intrinsically resistant to targeted agents. Chemotherapies could have also remodelled the TME in these patients via the induction of programmed death-ligand 1 (PD-L1) on CCA cells and increased anti-tumour activity of cytotoxic lymphocytes [24,25]. A triplet combination of chemotherapy with immune checkpoint inhibitors (ICIs) and targeted agents in CCA is an important avenue of clinical investigation that is currently underway in ongoing clinical trials (NCT06178445, NCT04430738). Such preliminary findings, albeit limited by sample size, support the rationale of favouring the use of targeted therapies over conventional chemotherapeutic regimens in the earlier-treatment line setting to optimise clinical outcomes for this challenging clinical population. Owing to the reduced toxicity associated with targeted therapy use, this treatment sequence would permit the retention of good performance status and preserve the quality of life to qualify such patients for further treatment in clinical trials or as part of standard-of-care treatment later in the course of their disease. The use of liquid biopsy methods to detect new resistant mutations among patients with refractory disease while on standard chemotherapy or previous targeted therapies can be instrumental in further targeted treatment selection, which could promote disease stabilisation and potentially improve quality of life. Our findings also highlight the potential role of pre-treatment surgery, which could contribute to improved overall survival in tandem with targeted therapies. Previous investigations have revealed an improvement in overall survival in CCA patients treated with adjuvant therapy following surgery [26,27]. Such improvements tend to increase especially amongst patients undergoing resection with high-risk features including T3/T4 tumours, positive lymph nodes and R0/R1 surgical margins [27,28]. Surgery promotes a decrease in tumour burden and limits the presence of intrinsically resistant subclones. A surgical intervention followed by the use of efficacious targeted agents could lead to improved disease control by delaying treatment resistance and promoting the elimination of micrometastatic disease near the surgical bed and lymph nodes. This approach could also delay the use of next-line systemic treatment and potentially contribute to improved overall survival. Surgical resection can also permit the acquisition of a larger tumour specimen, which can be used to determine the PD-L1 score and thereby promote consideration of ICIs in combination with targeted agents in future studies. However, the selection of such patients requires careful consideration of disease biology via molecular profiling results along with clinical factors in a multi-disciplinary setting. With UCLH serving as a regional centre for CCA patients, such an approach is strongly advocated by the well-established and experienced multi-disciplinary team at our institution. Recent investigations have also identified non-alcoholic steatohepatitis (NASH) as a risk factor for the development of intrahepatic CCA and reduced overall survival [29]. We did not observe NASH among patients evaluated in our study. This has implications for our study as the absence of NASH could have contributed to the improved overall survival noted among our CCA patients treated with futibatinib. Thus, an ideal treatment selection criterion would comprise intrahepatic CCA subjects without NASH but attaining R0/R1 resection status with FGFR2 aberrations who are then recommended for futibatinib treatment. However, the utility of such a treatment selection strategy warrants further confirmation in future studies.

Despite the encouraging activity of targeted agents, additional investigations are required to further improve treatment response and PFS and OS outcomes in this challenging clinical population. Future studies need to address the key issue of overcoming acquired resistance, which, in the case of pemigatinib and futibatinib, leads to hindrance of drug–target interactions due to steric effects, low binding affinity and inactive conformation binding [30]. Recent data involving the use of next-generation targeted agent tinengotinib have successfully demonstrated its remedial effect of overcoming FGFR2 kinase domain mutations following treatment failure with previous anti-FGFRs [30]. This led to a similar PFS (6.90 months) to FGFR-naïve patients in the FIGHT-202 trial [30]. Additional future opportunities to overcome acquired resistance involve the exploration of targeted agents in combination with other systemic agents, which can potentially mitigate the selective pressure on CCA cells and delay treatment progression, thereby resulting in a more prolonged treatment response in CCA patients. Such trials should not only consider the molecular profile of CCA but also account for the TME, which can be re-engineered via treatment combinations with immunotherapies. This can help prevent possible TME-driven treatment resistance. Finally, patient stratification is important in personalising the use of targeted agents in CCA. For instance, it would be ideal to not only identify additional clinical factors other than CCA surgical history to improve survival outcomes with targeted agents but also to identify FGFR-specific molecular subgroups that would derive the greatest clinical benefit from anti-FGFR use. Advances in computational technology, especially in the areas of artificial intelligence and deep machine learning, can further aid the discovery of such potential biomarkers and molecular drug targets in future studies [31]. 

## 5. Conclusions

Our data support the consideration of targeted agents for patients with advanced, metastatic cholangiocarcinoma. Patients treated with second-line targeted agents beyond 3.52 months are likely to attain improved survival outcomes, potentially due to the promotion of greater disease stabilisation and the achievement of a partial treatment response. Such a group of patients is most likely to benefit from a personalised medicine approach following genetic sequencing of their tumour specimens to target actionable molecular aberrations. Achieving a systemic response is important and likely contributes to favourable PFS and OS outcomes and may help delay switching treatment to more toxic chemotherapies associated with limited efficacy. These findings should be validated in additional prospective clinical trials. 

## Figures and Tables

**Table 1:** Baseline characteristics of evaluated patients (N = 56).

	Total Number of Subjects	
	N	%
**Age at CCA diagnosis (years)**		
Median	59	
Range	22–78	
Interquartile range	52–66	
**Gender**		
Male	23	41
Female	33	59
**Smoking Status**		
Never Smoker	13	23
Current Smoker	1	2
Former Smoker	11	20
Unknown	31	55
**ECOG Performance Status prior to commencing 1st targeted therapy**		
ECOG 0	5	9
ECOG 1	18	32
Unknown	33	59
**CCA subtype**		
Hilar	4	7
Intrahepatic	32	57
Extrahepatic	11	20
Unknown	9	16
**Stage at initial diagnosis**		
Localised	26	46
Metastatic	26	46
Unknown	4	8
**Anatomic site of metastasis prior to commencing 1st targeted therapy**		
Lung	17	30
Liver	36	64
Bone	5	9
Brain	1	2
Lymph node	18	32
**Prior treatment**		
Any CCA-related surgery	23	41
Radiation therapy	3	5
**Co-morbidities**		
Hypertension	11	20
Hypercholesterolemia	2	4
Transient ischemic attack	2	4
Supraventricular tachycardia	1	2
Atrial fibrillation	1	2
Asthma	3	5
Chronic Obstructive Pulmonary Disease	2	4
Hypothyroidism	3	5
Right-sided gynecomastia	1	2
Anxiety/mixed anxiety and depressive disorder	1	2
Low mood	1	2
Migraine	1	2
Alzheimer’s disease	1	2
Celiac disease	1	2
Inflammatory Bowel Syndrome	2	4
Bowel adhesions	1	2
Ulcerative colitis	1	2
Acid reflux	1	2
Barrett’s oesophagus	1	2
Type 1 diabetes mellitus	1	2
Type 2 diabetes mellitus	6	11
Controlled HIV	1	2
Chronic Hepatitis C	1	2
Chronic Kidney Disease (Stage 1)	1	2
Chronic periodontitis	1	2
Osteoarthritis	2	4
Osteoporosis	1	2
Vitamin D deficiency	1	2
Alopecia	1	2
Eczema	1	2
Benign Prostatic Hyperplasia	1	2

Abbreviation: CCA, cholangiocarcinoma; ECOG, Eastern Cooperative Oncology Group; HIV, Human Immunodeficiency Virus.

**Table 2:** Systemic treatment details of evaluated patients (N = 56).

	Total Number of Subjects	
	N	%
**Number of lines of systemic therapy**		
1st-line therapy	1	2
2nd-line therapy	25	45
3rd-line therapy	21	38
4th-line therapy	6	11
5th-line therapy	0	0
6th-line therapy	3	5
**1st-Line systemic therapy**	**56**	**100**
Chemotherapy	52	93
Chemotherapy + immunotherapy	3	5
Targeted agents	1	2
**2nd-Line systemic therapy**	**55**	**100**
Chemotherapy	20	36
Targeted agents	34	62
Targeted agents + chemotherapy	1	2
**3rd-Line systemic therapy**	**29**	**100**
Chemotherapy	10	34
Targeted agents	17	59
Targeted agents + chemotherapy	1	3
Targeted agents + immunotherapy	1	3
**4th-Line systemic therapy**	**9**	**100**
Chemotherapy	3	33
Targeted agents	6	67
**5th-Line systemic therapy**	**3**	**100**
Chemotherapy	3	100
**6th-Line systemic therapy**	**3**	**100**
Chemotherapy	1	33
Targeted agents	2	67
**Patients receiving 1st-line treatment (targeted agents) based on genomic profiling results**		
Yes	1	100
No	0	0
**Patients receiving 2nd-line treatment (targeted agents) based on genomic profiling results**		
Yes	32	91
No	3	9
**Patients receiving 3rd-line treatment (targeted agents) based on genomic profiling results**		
Yes	16	84
No	3	16
**Patients receiving 4th-line treatment (targeted agents) based on genomic profiling results**		
Yes	6	100
No	0	0
**Patients receiving 6th-line treatment (targeted agents) based on genomic profiling results**		
Yes	2	100
No	0	0

**Table 3:** Associations with PFS following 1st-, 2nd- and 3rd-line systemic agents.

	PFS (1st-Line Systemic Treatment)				PFS (2nd-Line Systemic Treatment)				PFS (3rd-Line Systemic Treatment)			
	Univariate HR (95% CI)	*p*-Value	Adjusted HR (95% CI) ^a^	*p*-Value	Univariate HR (95% CI)	*p*-Value	Adjusted HR (95% CI) ^a^	*p*-Value	Univariate HR (95% CI)	*p*-Value	Adjusted HR (95% CI) ^a^	*p*-Value
**Age** (<70 years vs. ≥70 years)	2.29 (0.78–6.76)	0.13	N/A	N/A	0.56 (0.23–1.37)	0.20	N/A	N/A	1.06 (0.31–3.67)	0.93	N/A	N/A
**Gender** (Male vs. Female)	0.91 (0.43–1.94)	0.82	N/A	N/A	0.85 (0.45–1.59)	0.61	N/A	N/A	1.16 (0.48–2.81)	0.75	N/A	N/A
**Smoking status** (Current or former smoker vs. never smoker)	1.73 (0.51–5.91)	0.38	N/A	N/A	1.06 (0.43–2.62)	0.91	N/A	N/A	1.19 (0.26–5.33)	0.82	N/A	N/A
**CCA subtype**												
Hilar	-	-	-	-	-	-	-	-	-	-	-	-
Intrahepatic	0.75 (0.17–3.31)	0.70	N/A	N/A	0.72 (0.25–2.10)	0.55	N/A	N/A	0.22 (0.02–1.88)	0.16	N/A	N/A
Extrahepatic	0.56 (0.11–3.07)	0.53	N/A	N/A	0.56 (0.17–1.89)	0.35	N/A	N/A	0.13 (0.01–1.37)	0.09	N/A	N/A
**FGFR status** (Pathogenic variant vs. wild type)	2.27 (0.99–5.22)	0.05	N/A	N/A	0.67 (0.35–1.29)	0.23	N/A	N/A	0.74 (0.30–1.83)	0.51	N/A	N/A
**BRCA status** (Pathogenic variant vs. wild type)	0.58 (0.23–1.46)	0.25	N/A	N/A	1.64 (0.76–3.51)	0.20	N/A	N/A	3.64 (1.25–10.57)	0.02	3.99 (0.91–17.53)	0.07
**Prior CCA-related surgical history**	0.98 (0.46–2.08)	0.96	N/A	N/A	1.06 (0.56–2.00)	0.86	N/A	N/A	1.12 (0.46–2.70)	0.81	N/A	N/A
**Best overall response on 2nd-line targeted therapy** (Responders vs. Non-Responders)	N/A	N/A	N/A	N/A	0.07 (0.02–0.32)	<0.001	0.09 (0.02–0.43)	0.002	N/A	N/A	N/A	N/A
**Nature of 2nd-line targeted therapy** (Futibatinib vs. other targeted therapies)	N/A	N/A	N/A	N/A	0.43 (0.20–0.96)	0.04	0.11 (0.03–0.49)	0.004	N/A	N/A	N/A	N/A
**Duration of 2nd-line targeted therapy** (>3.52 months vs. ≤3.52 months)	N/A	N/A	N/A	N/A	N/A ^b^	N/A ^b^	N/A ^b^	N/A ^b^	N/A	N/A	N/A	N/A
**Best overall response on 3rd-line targeted therapy** (Responders vs. Non-Responders)	N/A	N/A	N/A	N/A	N/A	N/A	N/A	N/A	0.18 (0.05–0.73)	0.02	0.18 (0.02–0.67)	0.02
**Nature of 3rd-line targeted therapy** (Futibatinib vs. other targeted therapies)	N/A	N/A	N/A	N/A	N/A	N/A	N/A	N/A	1.79 (0.46–6.91)	0.40	N/A	N/A
**Duration of 3rd-line targeted therapy** (>4.37 months vs. ≤4.37 months)	N/A	N/A	N/A	N/A	N/A	N/A	N/A	N/A	0.06 (0.007–0.53)	0.01	0.06 (0.007–0.57)	0.01

^a^ Adjusted analysis by age, gender and CCA subtype. ^b^ A CoxPH analysis is not applicable in this instance due to presence of the Hauck–Donner effect. Abbreviations: BRCA, BReast CAncer gene; CCA, cholangiocarcinoma; CI, confidence interval; FGFR, Fibroblast Growth Factor Receptor gene; HR, Hazard Ratio; N/A, Not Applicable; PFS, progression-free survival.

## Data Availability

The original contributions presented in this study are included in the article/Supplementary Material; further inquiries can be directed to the corresponding author.

## Supplementary Materials

The following supporting information can be downloaded at https://www.mdpi.com/article/10.3390/cancers16040697/s1, Supplementary Figure S1: Median duration of clinical benefit following systemic therapy in a 1st line (AB), 2nd line (B) and 3rd line setting (C). Clinical benefit refers to patients achieving complete response, partial response or stable disease as their best overall treatment response.; Supplementary Figure S2: Associations between best overall response on 2nd line targeted agents and PFS (A) and OS (B), respectively. Associations were also evaluated between targeted treatment type in a 3rd line treatment setting and PFS (C) and OS (D), respectively. Patients achieving partial response were classified as responders whereas subjects attaining stable disease or progressive disease as their best overall response were categorized as non-responders.; Supplementary Figure S3: Associations between targeted treatment type in a 2nd line setting and PFS (A) and OS (B), respectively. Associations were also evaluated between targeted treatment type in a 3rd line setting and PFS (C) and OS (D), respectively. Other targeted therapies did not include other systemic agent combinations such as chemotherapy and immunotherapeutic agents.; Supplementary Figure S4: Pre-treatment baseline scan of Subject # 6 from May 2020 demonstrates 1 liver metastases with largest axial measurement of 11.3 cm (A). Patient achieved partial response in August 2020 while on 2nd line futibatinib which demonstrated the greatest regression in liver tumour volume in May 2021 limited to 62.0 mm (B).; Supplementary Figure S5: Pre-treatment baseline scan of Subject # 46 from May 2020 demonstrates 1 liver metastases with largest axial measurement of 11.0 cm (A). Patient achieved partial response in October 2020 while on 4th line futibatinib which demonstrated the greatest regression in liver tumour volume at this timepoint which was limited to 61.4 mm (B). Supplementary Table S1: Assessment of CCA lesions based on the RECIST 1.1 criteria.; Supplementary Table S2: Systemic treatment types stratified by treatment lines among evaluated patients (N = 56).; Supplementary Table S3: Associations with OS.; Supplementary Table S4: Best overall response following 1st line treatment with systemic agents (N = 56).; Supplementary Table S5: Best overall response following 2nd line treatment with chemotherapeutic regimens and targeted agents (N = 55).; Supplementary Table S6: Best overall response following 3rd line treatment with chemotherapeutic regimens and targeted agents (N = 29).
