# Supplementary material for: Comprehensive Examination of Cholangiocarcinoma Patients Treated with Novel Targeted Therapies after Extended Molecular Profiling on Liquid Biopsies
Source: Cancers (Basel). 2024 Feb 6;16(4):697. doi: 10.3390/cancers16040697 (PMC10886944; doi:10.3390/cancers16040697)
Supplement: Supplementary file 1 [file cancers-16-00697-s001.zip › Supplementary Figures.pdf]

A

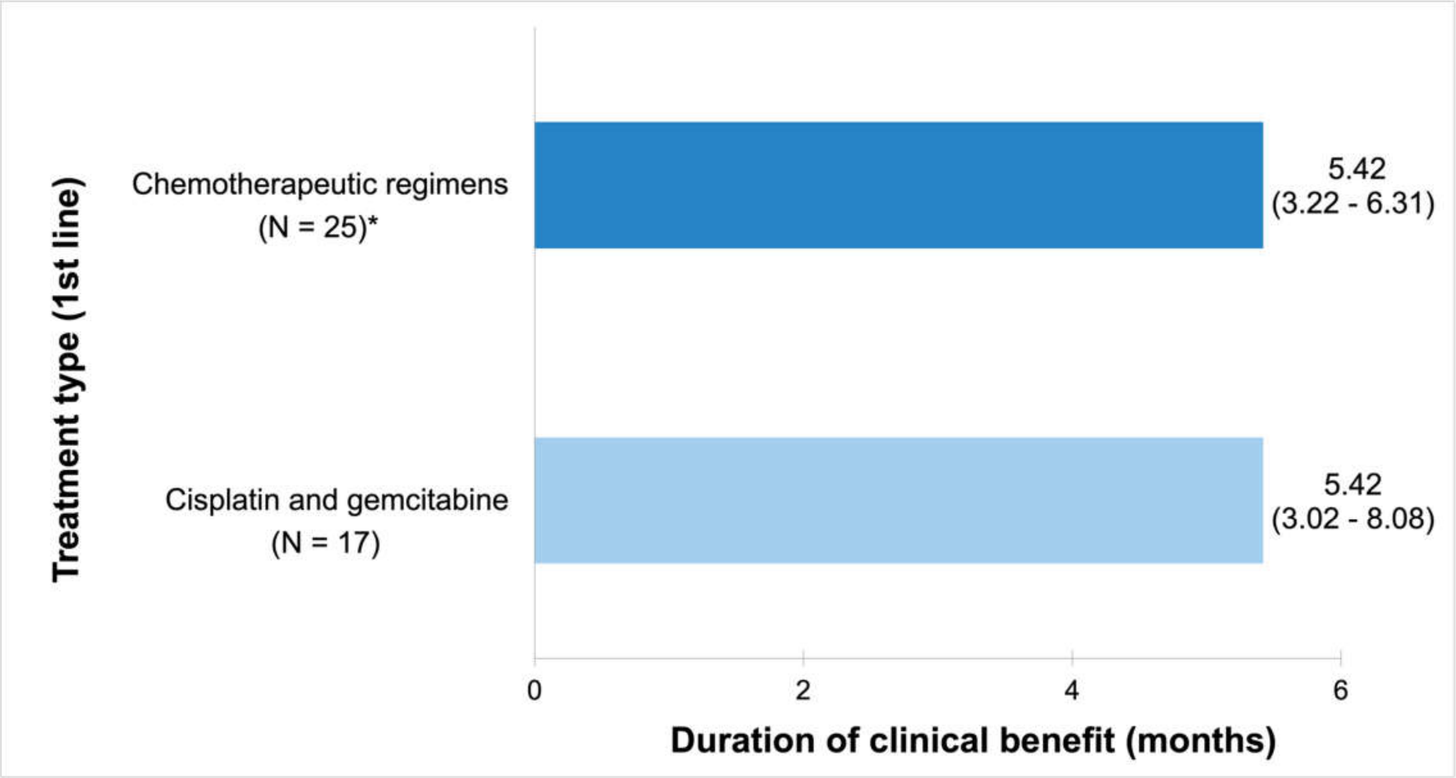
Treatment type (1st line)
Chemotherapeutic regimens
(N = 25)*
5.42
(3.22 - 6.31)
Cisplatin and gemcitabine
(N = 17)
5.42
(3.02 - 8.08)
0
2
4
6
Duration of clinical benefit (months)

B

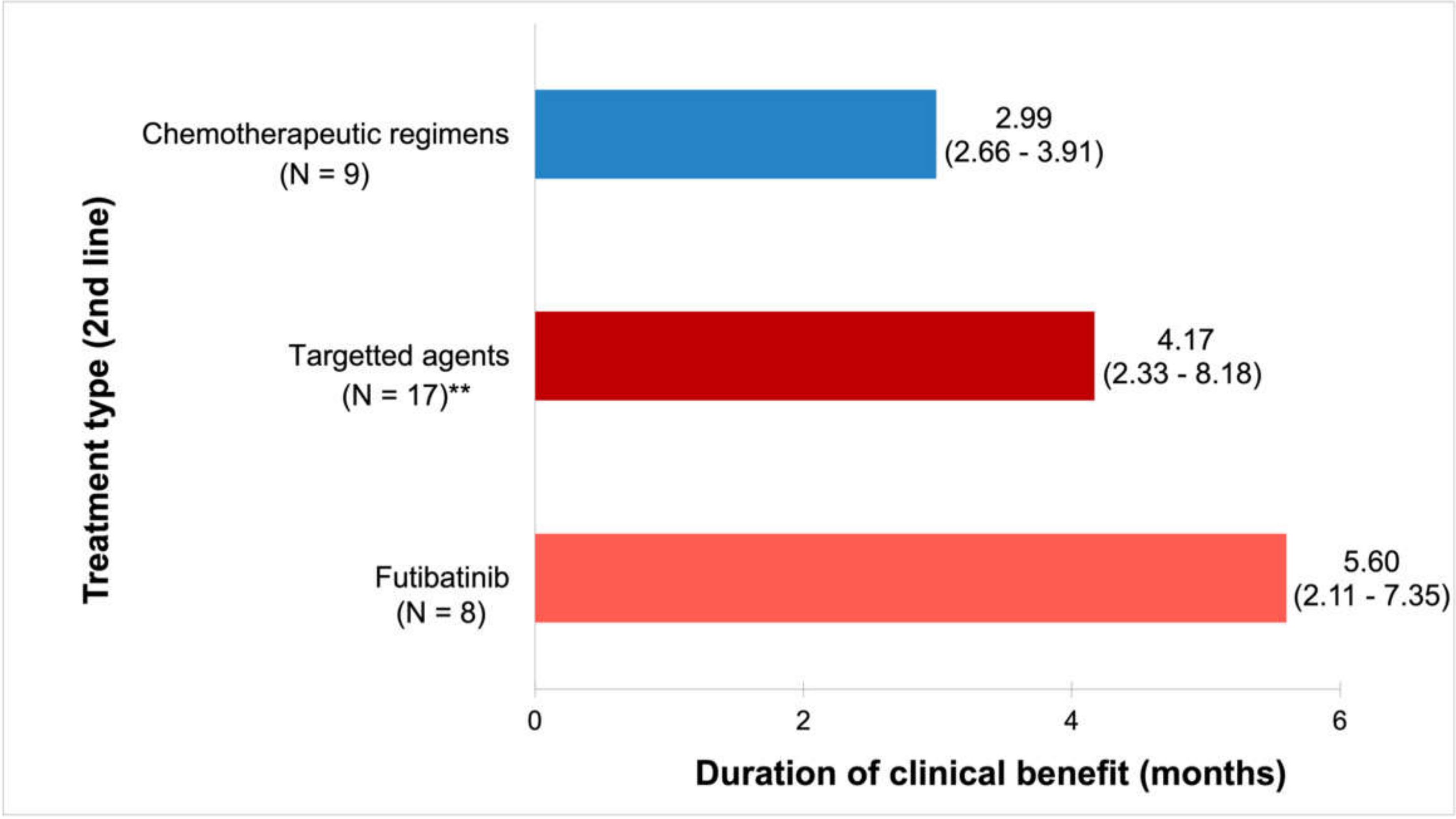
Treatment type (2nd line)
Chemotherapeutic regimens
(N = 9)
2.99
(2.66 - 3.91)
Targetted agents
(N = 17)**
4.17
(2.33 - 8.18)
Futibatinib
(N = 8)
5.60
(2.11 - 7.35)
0
2
4
6
Duration of clinical benefit (months)

C

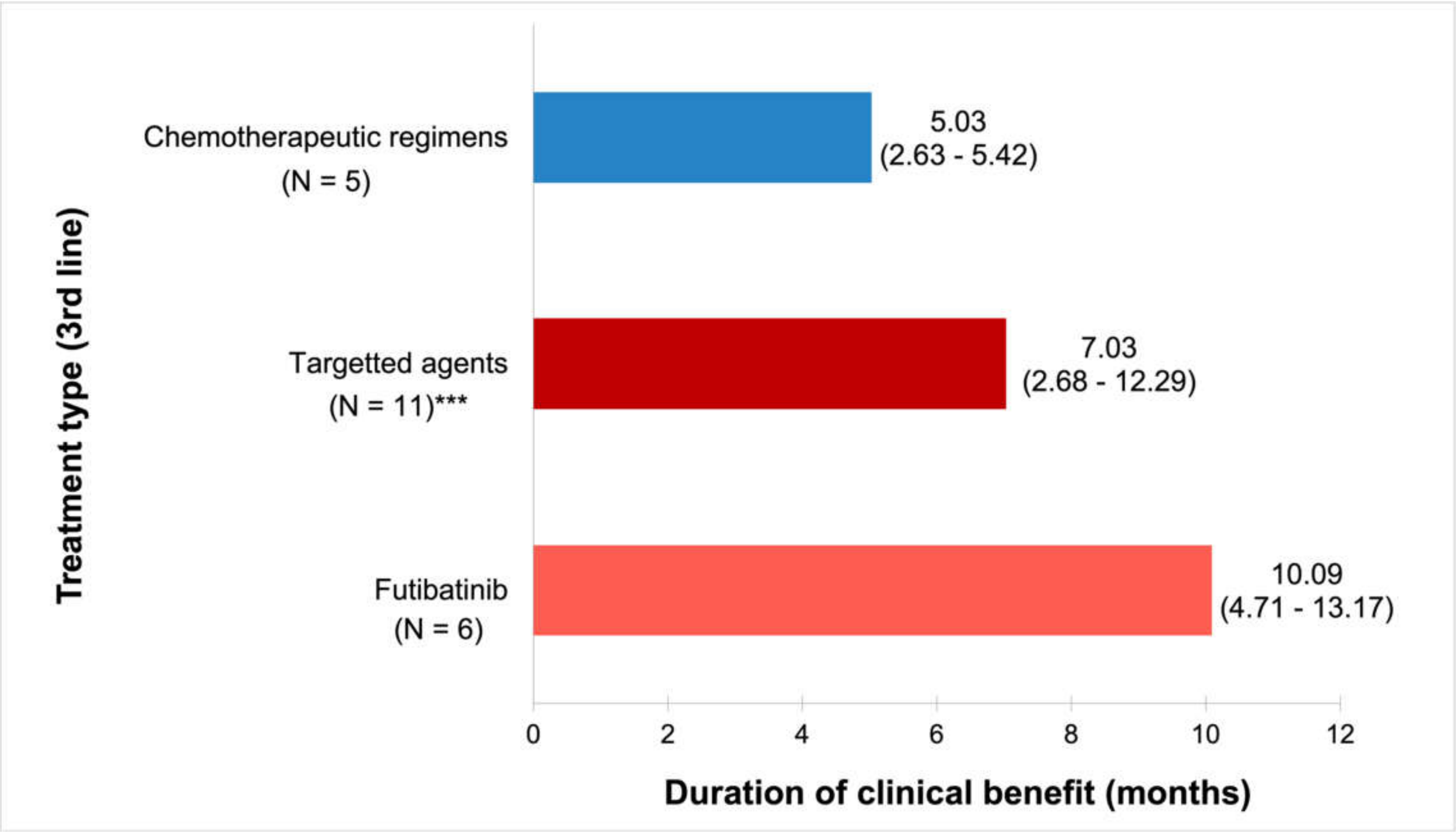

Supplementary Figure S1: Median duration of clinical benefit following systemic therapy in a 1st line (AB), 2nd line (B) and 3rd line setting (C). Clinical benefit refers to patients achieving complete response, partial response or stable disease as their best overall treatment response.

*** Includes 2 patients receiving immunotherapy and chemotherapy.**

**** Includes 1 patient receiving targeted agent and chemotherapy.**

***** Includes 1 patient receiving targeted agent and immune checkpoint inhibitor and 1 patient receiving targeted agent and chemotherapy.**

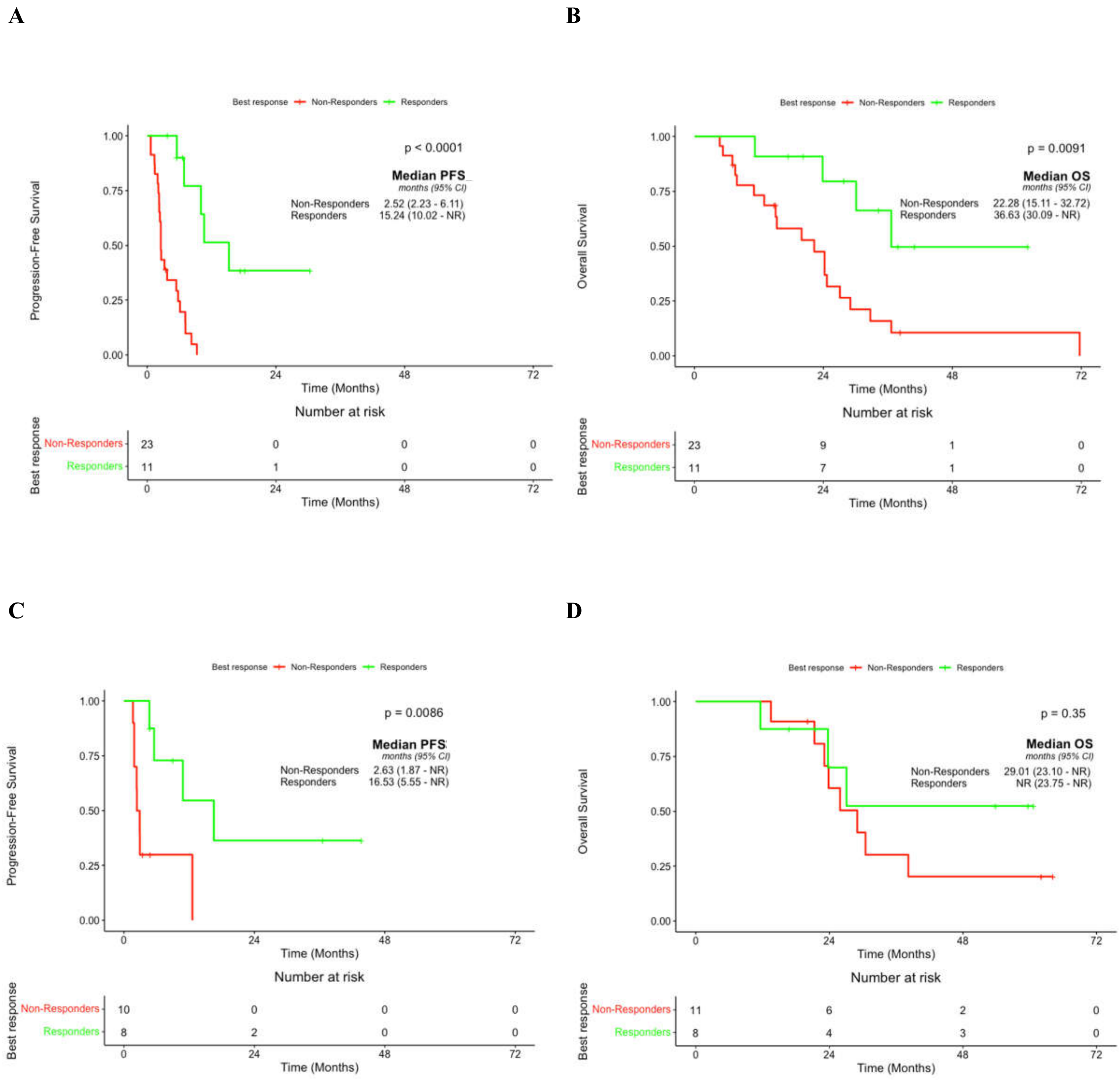

Supplementary Figure S2: Associations between best overall response on 2nd line targeted agents and PFS (A) and OS (B), respectively. Associations were also evaluated between **targeted treatment type in a 3rd line treatment setting and PFS (C) and OS (D), respectively. Patients achieving partial response were classified as responders whereas subjects attaining stable disease or progressive disease as their best overall response were categorized as non-responders.**

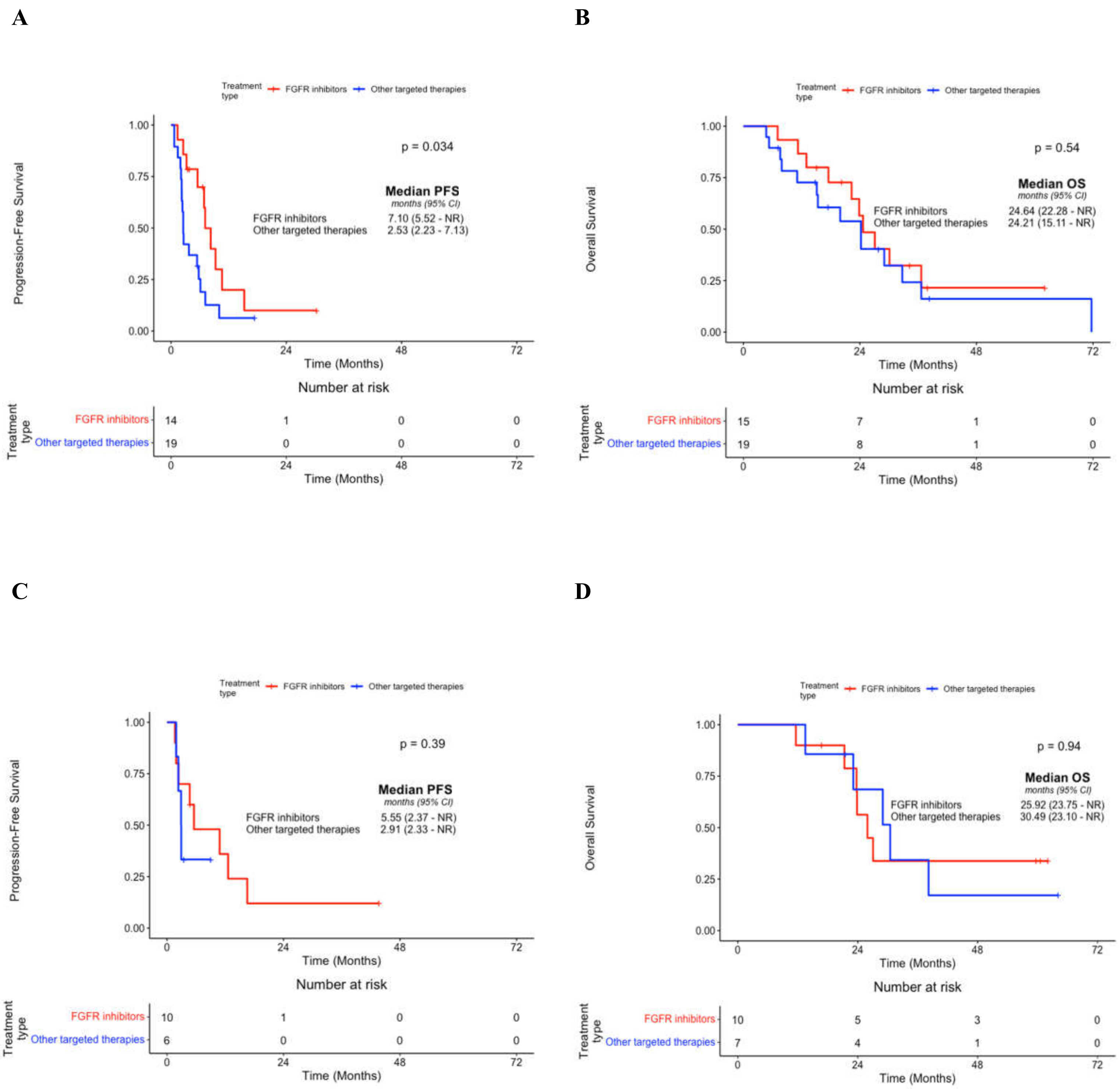

Supplementary Figure S3: Associations between targeted treatment type in a 2nd line setting and PFS (A) and OS (B), respectively. Associations were also evaluated between **targeted treatment type in a 3rd line setting and PFS (C) and OS (D), respectively. Other targeted therapies did not include other systemic agent combinations such as chemotherapy and immunotherapeutic agents.**

A

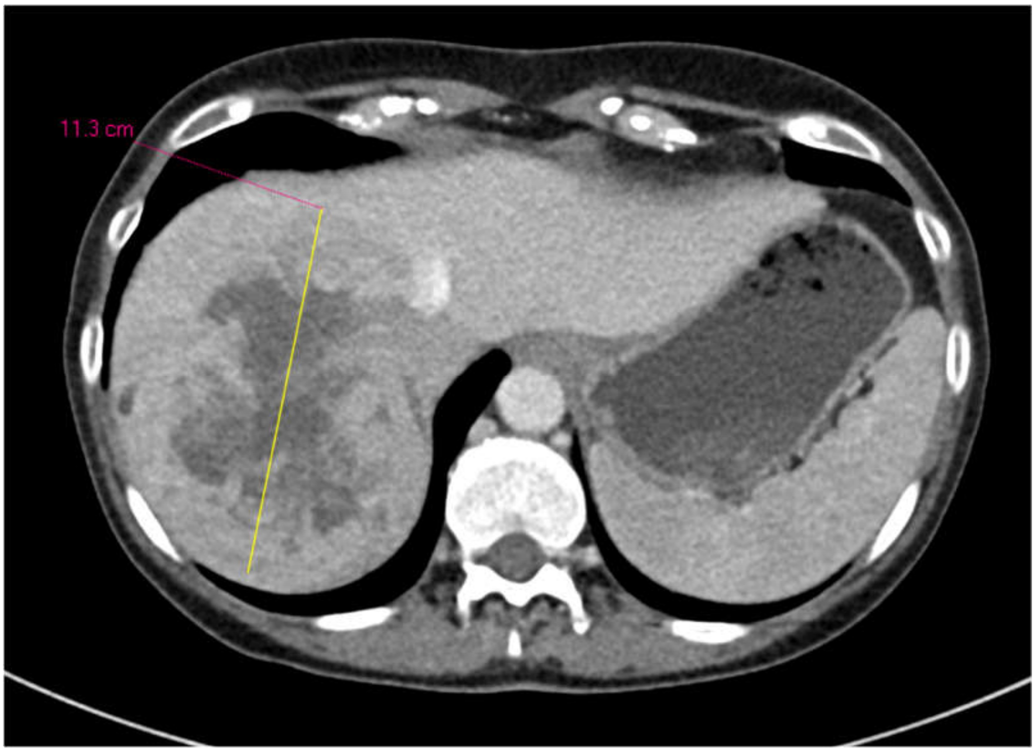

B

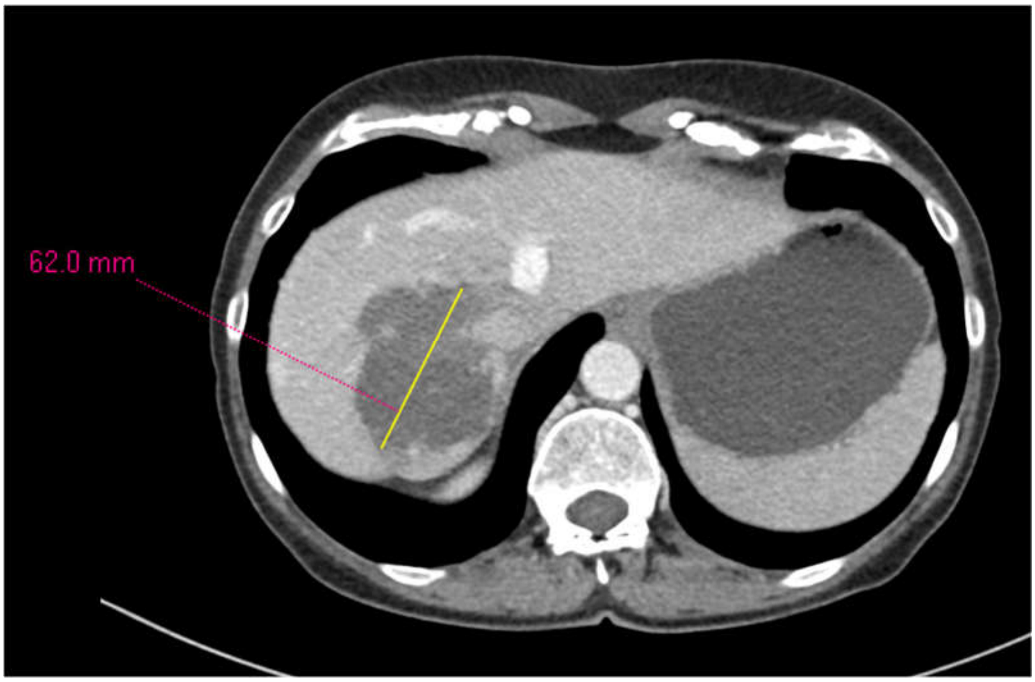

Supplementary Figure S4: Pre-treatment baseline scan of Subject # 6 from May 2020 demonstrates 1 liver metastases with largest axial measurement of 11.3 cm (A). Patient achieved partial response in August 2020 while on 2nd line futibatinib which demonstrated the greatest regression in liver tumour volume in May 2021 limited to 62.0 mm (B).

A

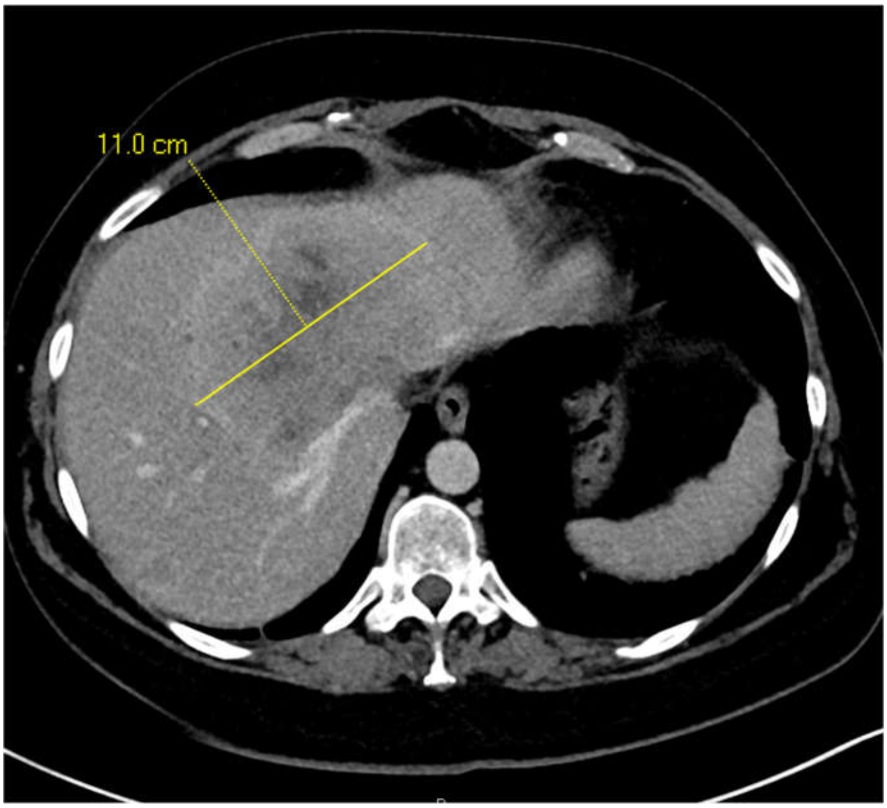

B

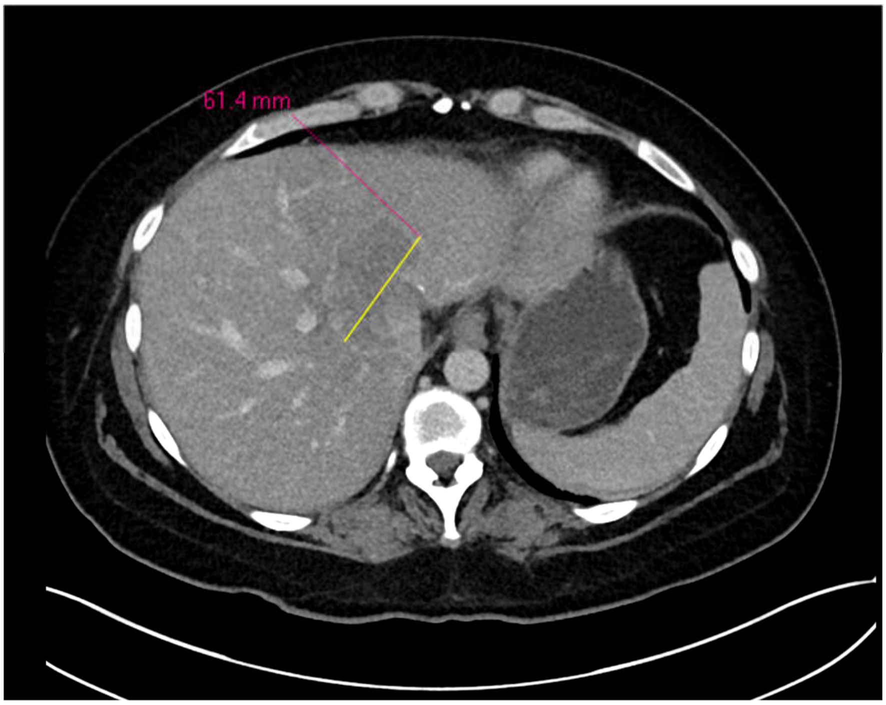

Supplementary Figure S5: Pre-treatment baseline scan of Subject # 46 from May 2020 demonstrates 1 liver metastases with largest axial measurement of 11.0 cm (A). Patient achieved partial response in October 2020 while on 4th line futibatinib which demonstrated the greatest regression in liver tumour volume at this timepoint which was limited to 61.4 mm (B).
