# Supplementary material for: Comprehensive Examination of Cholangiocarcinoma Patients Treated with Novel Targeted Therapies after Extended Molecular Profiling on Liquid Biopsies
Source: Cancers (Basel). 2024 Feb 6;16(4):697. doi: 10.3390/cancers16040697 (PMC10886944; doi:10.3390/cancers16040697)
Supplement: Supplementary file 1 [file cancers-16-00697-s001.zip › Supplementary Tables 31 Jan 24.pdf]

Supplementary Tables

Supplementary Table S1: Assessment of CCA lesions based on the RECIST 1.1 criteria.

Response	Target lesions	Non-target	New Lesions
Complete response	Disappearance of all target lesions	Disappearance of all non-target lesions	No development of new lesions
	Lymph node axis < 10 mm	Lymph node axis < 10 mm	
Partial response	≥ 30% decrease in sum of longest diameter from baseline (≥ 4 weeks)	No progression	No development of new lesions
Progressive disease	≥ 20% increase in sum of longest diameter from Nadir with an absolute sum of diameters increase ≥ 5 mm	Unequivocal progression in lesion size	Unequivocal development of new lesions
Stable disease	Neither partial response nor progressive disease with the Nadir as reference point	Persistence of one or more non-target lesions	No development of new lesions

Supplementary Table S2: Systemic treatment types stratified by treatment lines among evaluated patients (N = 56).

	Total number of subjects	
	N	%
**1st line systemic therapy**	**56**	**100**
Chemotherapy		
Cisplatin and gemcitabine	38	68
Capecitabine	5	9
Capecitabine and gemcitabine	2	4
Carboplatin and etoposide	2	4
Carboplatin and gemcitabine	2	4
Oxaliplatin and gemcitabine	1	2
Paclitaxel and gemcitabine	1	2
Cisplatin and pemetrexed	1	2
Chemotherapy and immunotherapy		
Cisplatin, gemcitabine and Arginase inhibitor	1	2
Cisplatin, gemcitabine and Durvalumab	2	4
Targeted therapy		
Ivosidenib	1	2
**2nd line systemic therapy**	**55**	**100**
Chemotherapy		
Capecitabine	2	4
Carboplatin and gemcitabine	2	4
Cisplatin and gemcitabine	4	7
Cisplatin, gemcitabine and TAS-102	1	2
Epirubicin, oxaliplatin and capecitabine	1	2
FOLFOX	9	16
Unknown 2nd line chemotherapeutic drug	1	2
Targeted therapy		
Futibatinib	11	20
Regorafenib	9	16
Olaparib	7	13

Derazantinib	3	5
Zanidatamab	2	4
Erdafitinib	1	2
Ivosidenib	1	2
Targeted therapy and chemotherapy		
Encorafenib and cetuximab	1	2
**3rd line systemic therapy**	**29**	**100**
Chemotherapy		
FOLFOX	7	24
Cisplatin and gemcitabine	3	10
Targeted therapy		
Futibatinib	7	24
Regorafenib	5	17
Derazantinib	3	10
Pertuzumab and trastuzumab	1	3
Zanidatamab	1	3
Targeted therapy and chemotherapy		
Encorafenib and cetuximab	1	3
Targeted therapy and immunotherapy		
Bintrafusp alfa	1	3
**4th line systemic therapy**	**9**	**100**
Chemotherapy		
FOLFOX	1	11
Cisplatin and gemcitabine	1	11
FOLFIRI	1	11
Targeted therapy		
Futibatinib	3	33
Regorafenib	3	33
**5th line systemic therapy**	**3**	**100**
Chemotherapy		
Capecitabine	1	33
Gemcitabine	1	33
Cisplatin and gemcitabine	1	33

6th line systemic therapy	3	100
Chemotherapy		
Oxaliplatin and Capecitabine	1	33
Targeted therapy		
Crizotinib	1	33
Ivosidenib	1	33

Supplementary Table S3: Associations with OS.

	OS			
	**Univariate HR (95% CI)**	**p-value**	**Adjusted HR (95% CI)[a]**	**p-value**
**Age** (< 70 years vs ≥ 70 years)	0.54 (0.25 – 1.21)	0.14	N/A	N/A
**Gender** (Male vs Female)	0.61 (0.30 – 1.23)	0.17	N/A	N/A
**Smoking status** (Current or former smoker vs never smoker)	0.79 (0.30 – 2.14)	0.65	N/A	N/A
**CCA subtype**				
Hilar	-	-	-	-
Intrahepatic	2.09 (0.45 – 9.80)	0.35	N/A	N/A
Extrahepatic	1.03 (0.19 – 5.49)	0.97	N/A	N/A
**FGFR status** (Pathogenic variant vs wild type)	1.12 (0.56 – 2.23)	0.75	N/A	N/A
**BRCA status** (Pathogenic variant vs wild type)	1.44 (0.64 – 3.25)	0.38	N/A	N/A
**Prior CCA related surgical history**	0.39 (0.18 – 0.82)	0.01	0.30 (0.11 – 0.80)	0.02

**Total number of systemic treatment lines** (> 2 lines vs ≤ 2 lines)	0.60 (0.30 – 1.18)	0.14	N/A	N/A
**Best overall response on 2nd line targeted therapy** (Responders vs Non-Responders)	0.26 (0.09 – 0.77)	0.02	0.23 (0.07 – 0.75)	0.02
**Nature of 2nd line targeted therapy** (Futibatinib vs other targeted therapies)	0.77 (0.34 – 1.76)	0.54	N/A	N/A
**Duration of 2nd line targeted therapy** (> 3.52 months vs ≤ 3.52 months)	0.18 (0.07 – 0.46)	< 0.001	0.02 (0.002 – 0.14)	< 0.001
**Best overall response on 3rd line targeted therapy** (Responders vs Non-Responders)	0.54 (0.14 – 2.02)	0.36	N/A	N/A
**Nature of 3rd line targeted therapy** (Futibatinib vs other targeted therapies)	1.05 (0.32 – 3.46)	0.94	N/A	N/A
**Duration of 3rd line targeted therapy** (> 4.37 months vs ≤ 4.37 months)	0.21 (0.04 – 0.96)	0.04	0.23 (0.03 – 1.68)	0.15

[a] Adjusted analysis by age, gender and CCA subtype.
Abbreviations: BRCA, BReast CAncer gene; CCA, Cholangiocarcinoma; CI, Confidence Interval; FGFR, Fibroblast Growth Factor Receptor gene; HR, Hazard Ratio; N/A, Not Applicable; OS, Overall Survival.

Supplementary Table S4: Best overall response following 1st line treatment with systemic agents (N = 56).

	**Total number of subjects**	
	**N**	**%**
**Response status on chemotherapeutic regimens**		
Complete response	1	2
Partial response	13	23
Stable disease	14	25
Progressive disease	15	27
Unevaluable	9	16
**Response status on chemo-immunotherapeutic regimens**		
Stable disease	2	4
Progressive disease	1	2

**Response status on targeted agents**		
Stable disease	1	2

Supplementary Table S5: Best overall response following 2nd line treatment with chemotherapeutic regimens and targeted agents (N = 55).

	**Total number of subjects**	
**Response status on chemotherapeutic regimens**	**N**	**%**
Partial response	5	9
Stable disease	7	13
Progressive disease	5	9
Unevaluable	3	5
**Response status on targeted agents**		
Partial response	10	18
Stable disease	8	15
Progressive disease	15	27
Unevaluable	1	2
**Response status on targeted therapy and chemotherapy**		
Partial response	1	2

Supplementary Table S6: Best overall response following 3rd line treatment with chemotherapeutic regimens and targeted agents (N = 29).

	Total number of subjects	
**Response status on chemotherapeutic regimens**	**N**	**%**
Partial response	2	7
Stable disease	3	10
Progressive disease	5	17
**Response status on targeted agents**		
Partial response	7	24
Stable disease	3	10
Progressive disease	7	24
**Response status on targeted agent and chemotherapy**		
Stable disease	1	3
**Response status on targeted agent and immunotherapy**		
Partial response	1	3
